# CDK12 and PAK2 as novel therapeutic targets for human gastric cancer

**DOI:** 10.7150/thno.46137

**Published:** 2020-05-15

**Authors:** Hui Liu, Seung Ho Shin, Hanyong Chen, Tingting Liu, Zhi Li, Yamei Hu, Fangfang Liu, Chengjuan Zhang, Dong Joon Kim, Kangdong Liu, Zigang Dong

**Affiliations:** 1 Department of Pathophysiology, School of Basic Medical Sciences, Academy of Medical Science, College of Medicine, Zhengzhou University, Zhengzhou, Henan, 450001, China.; 2 China-US (Henan) Hormel Cancer Institute, No.127, Dongming Road, Jinshui District, Zhengzhou, Henan, 450008, China; 3 Department of Digestive, The Affiliated Cancer Hospital of Zhengzhou University, Henan Cancer Hospital, No.127, Dongming Road, Jinshui District, Zhengzhou, Henan, 450008, China.; 4 The Hormel Institute, University of Minnesota, Austin, MN 55912, USA.; 5 Department of Food and Nutrition, Gyeongsang National University, Jinju 52828, Republic of Korea

**Keywords:** gastric cancer, CDK12, PAK2, phosphorylation, procaterol

## Abstract

**Background:** Gastric cancer remains the second leading cause of cancer-related death, and the third in mortality due to lack of effective therapeutic targets for late stage cancer patients. This study aims to identify potential druggable target biomarkers as potential therapeutic options for patients with gastric cancer.

**Methods:** Immunohistochemistry of human gastric tumor tissues was conducted to determine the expression level of cyclin-dependent kinase 12 (CDK12). Multiple *in vitro* and *in vivo* assays such as RNAi, mass spectrometry, computer docking models, kinase assays, cell xenograft NU/NU mouse models (CDXs) and patient-derived xenograft NOD/SCID mouse models (PDXs) were conducted to study the function and molecular interaction of CDK12 with p21 activated kinase 2 (PAK2), as well as to find CDK12 inhibitors as potential treatment options for human gastric cancer.

**Results:** Here we identified that CDK12 is a driver gene in human gastric cancer growth. Mechanistically, CDK12 directly binds to and phosphorylates PAK2 at T134/T169 to activate MAPK signaling pathway. We further identified FDA approved clinical drug procaterol can serve as an effective CDK12 inhibitor, leading to dramatic restriction of cancer cell proliferation and tumor growth in human gastric cancer cells and PDXs.

**Conclusions:** Our data highlight the potential of CDK12/PAK2 as therapeutic targets for patients with gastric cancer, and we propose procaterol treatment as a novel therapeutic strategy for human gastric cancer.

## Introduction

According to the global cancer statistics in 2018, gastric cancer is the second leading cause of cancer death and third mortality based death 1, 2. The 5-year survival rate of gastric cancer remains lower than 30%. The main reason for this low survival rate is the lack of effective target proteins and their inhibitors for late stage gastric cancer treatment 3, 4.

Cyclin dependent kinase (CDK) 12 is a member of CDK family that plays important roles in a variety of diseases and tumorigenesis 5. There are 20 members in the CDK family (CDK 1-20), and several CDK inhibitors have been developed as anti-cancer drugs 6. CDK12 participates in all kinds of processes including cell cycle, transcription regulation, and DNA repair 7-9. CDK12 is known to phosphorylate the large subunit of RNA polymerase II (POLR2A) at the C-terminal domain (CTD), as well as translation factor 4E-BP1 7, 10, 11, thereby regulating transcription elongation and translation of a subset of mRNAs for cell division 12. Despite the efforts to identify CDK12 substrates 13, non-CTD substrates of CDK12 and the consequences of their phosphorylation remain uncertain. Moreover, the role of CDK12 in cancer is contradictory and unclear 14-21. Thus, elucidating the functional role and molecular activities of CDK12 in gastric cancer is worthy of investigation.

The p21 activated kinase 2 (PAK2) is a member of the PAK family serine/threonine kinases that function as downstream substrates of Rho family GTPases, Rac and CDC42 22, 23. Six members of PAKs (PAK 1-6) 24, 25 are positioned at an intersection of multiple hallmarks including almost all of the cell processes: proliferation, mitosis, survival, apoptosis, motility and angiogenesis in different cancer types 26-29. PAK2 regulates cell senescence and organismal aging by regulating gene expression and histone 3.3 (H3.3) nucleosome assemble 30. Most importantly, PAK2 has become a promising target for cancer prevention and might be associated with advanced tumor progression and poor prognosis in gastric cancer 31-33. The aim of this study is to identify and characterize the therapeutic potential and related mechanisms of CDK12 in human gastric cancer progression and find an effective CDK12 inhibitor for the treatment of gastric cancer.

## Results

### CDK12 is overexpressed in human gastric tumors and CDK12 knock-down suppresses cancer cell growth

**Figure 1:**
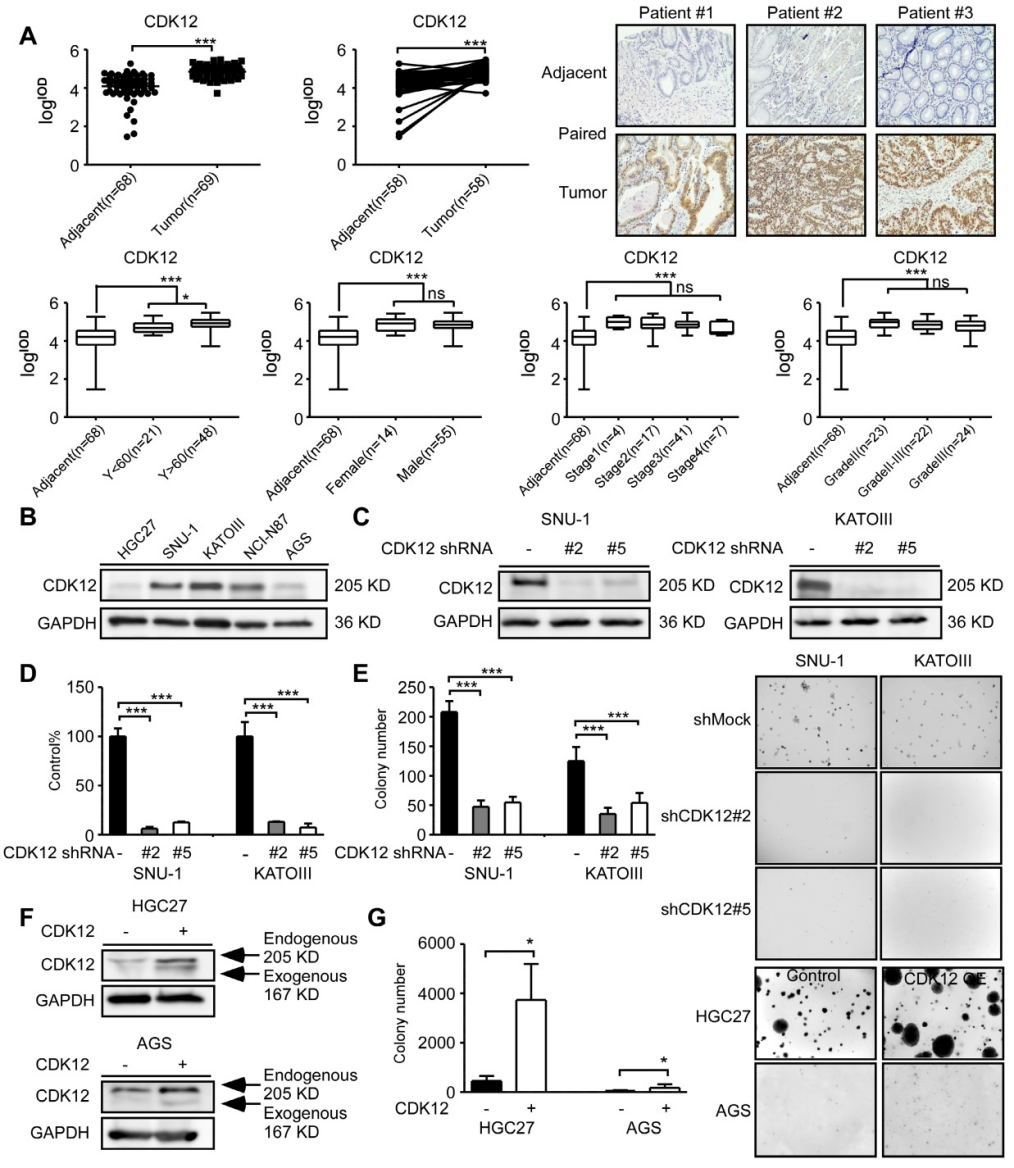
** CDK12 is a potential therapeutic target for human gastric cancer.** A. The expression of CDK12 was examined by immunohistochemistry (IHC) staining using a gastric tumor array. The upper panels show quantification of all samples and paired samples (n=58). The lower panels show expression of CDK12 in patients with different age, gender, clinical stages and tumor grades. The representative photographs of each group of paired samples are shown. 100 × magnification. B. The expression of CDK12 in human gastric cancer cell lines: HCG27, AGS, SNU-1, KATOIII, and NCI-N87 were evaluated by western blotting (WB). C. The level of CDK12 knock-down by lentiviral transduction (shMock, shCDK12#2 and shCDK12#5) was measured by western blotting in SNU-1 and KATOIII cell lines. D. Cell viability was measured after CDK12 knock-down by MTT (72 h after seeding cells) assay. E. Colony number was checked after CDK12 knocking down by anchorage-independent colony formation assay, representative photographs are shown. F. Protein levels were detected by WB after overexpression of CDK12 or control. G. Cell proliferation was measured by colony formation assay after overexpression of CDK12 or control, representative images are shown. Data represent means ±SD. P<0.05: *, p<0.01: **, p<0.001: ***. Significance determined by two-tailed Student's t test.

To measure CDK12 expression level in human gastric tumors, we performed immunohistochemistry (IHC) staining in human gastric tumor and adjacent tissues. CDK12 level was significantly higher in tumor tissues compared with adjacent tissues (Figure 1A, upper panel). The representative images are shown in paired tissues. While the expression level is higher in patients older than 60 years than those younger than 60 years, but there was no significant difference between tumors across different gender, clinical stages or grades (Figure 1A, lower panel). The information of cancer patients was shown in Table 1. We also evaluated CDK12 expression in human gastric cancer cell lines. Among five gastric cancer cell lines, SNU-1, KATOIII and NCI-N87 showed a relatively high expression of CDK12 (Figure 1B). Then, we tested different short hairpin (sh)RNAs targeting CDK12, to generate the stable CDK12 knockdown cells (Figure 1C). The cell viability assay and soft agar colony formation assay showed that knock-down of CDK12 dramatically inhibited gastric cancer viability and anchorage-independent growth (Figure 1D-E). We observed an increase in colony formation after overexpression of CDK12 in HGC27 and AGS cells (Figure 1F-G). These results suggest that CDK12 is overexpressed in human gastric tumors and plays an important role in cancer cell growth.

### Knock-down of CDK12 causes G2 phase arrest and suppresses gastric cancer cell growth *in vivo*

**Figure 2:**
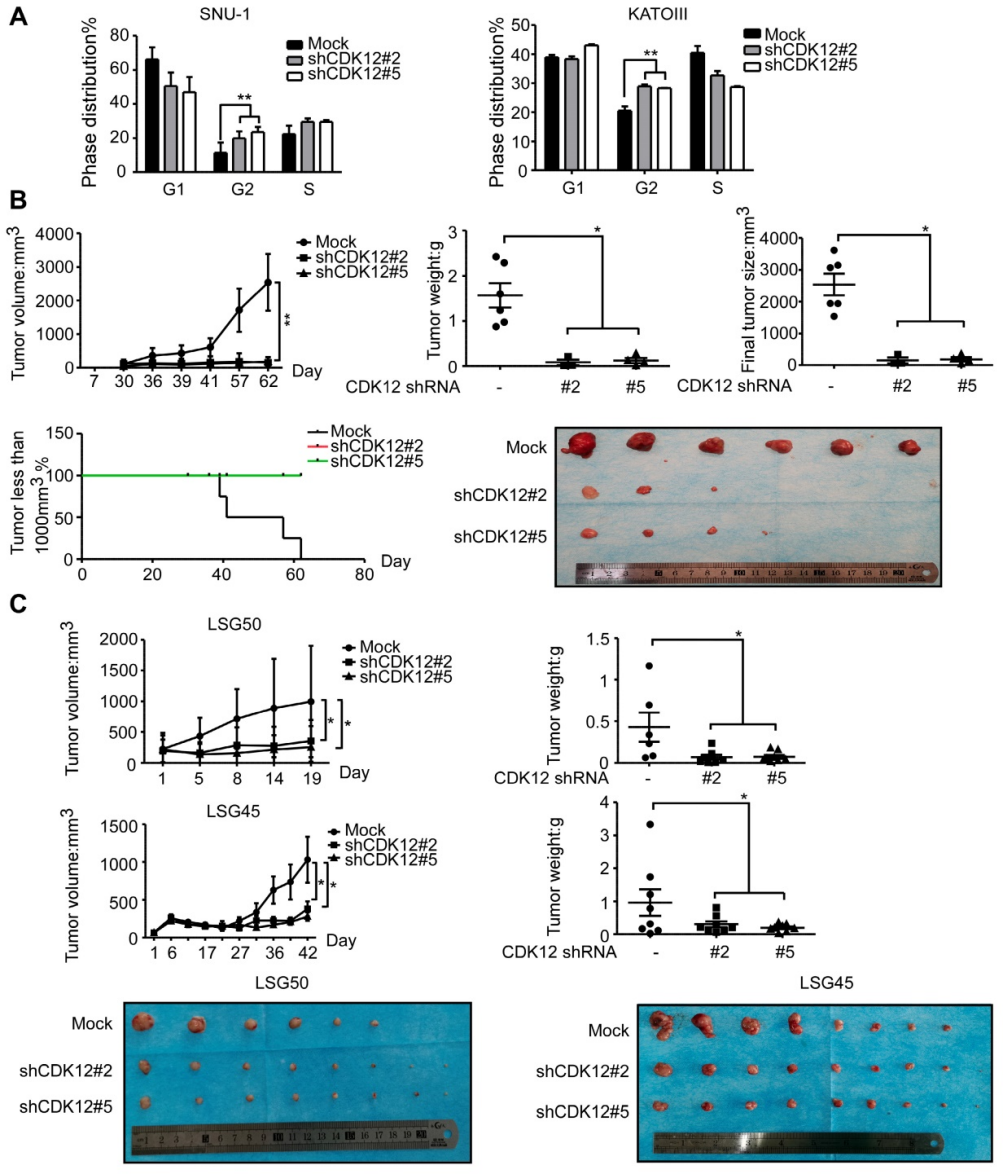
** Knock-down of CDK12 induces cell cycle arrest and delays tumor progression *in vivo*.** A. Cell cycle was detected by flow cytometry after CDK12 knock-down in SNU-1 and KATOIII cells 24 h after seeding the cells. B. Longitudinal tumor volume, tumor weight, final tumor size and percentage of mice with tumors less than 1000 mm^3^ at the endpoint were measured after the inoculation of shMock, shCDK12#2 and shCDK12#5 SNU-1 cells into NU/NU mice. Tumor size was measured every 3-7 days, and the volume was calculated by V = (length) × (width) × (height). The tumors in each group are shown. C. Longitudinal tumor volume and tumor weight were measured after injecting lentivirus (shMock, shCDK12#2 and shCDK12#5) directly into patient-derived xenografts (PDX) tumors at ~ 200 mm^3^. Two different cases (upper panels: LSG50, lower panels: LSG45) were tested. Tumor weight was measured at the endpoints. The tumors in each case are shown. Data represent means ±SD. P<0.05: *, p<0.01: **, p<0.001: ***. Significance determined by two-tailed Student's t test.

**Figure 3:**
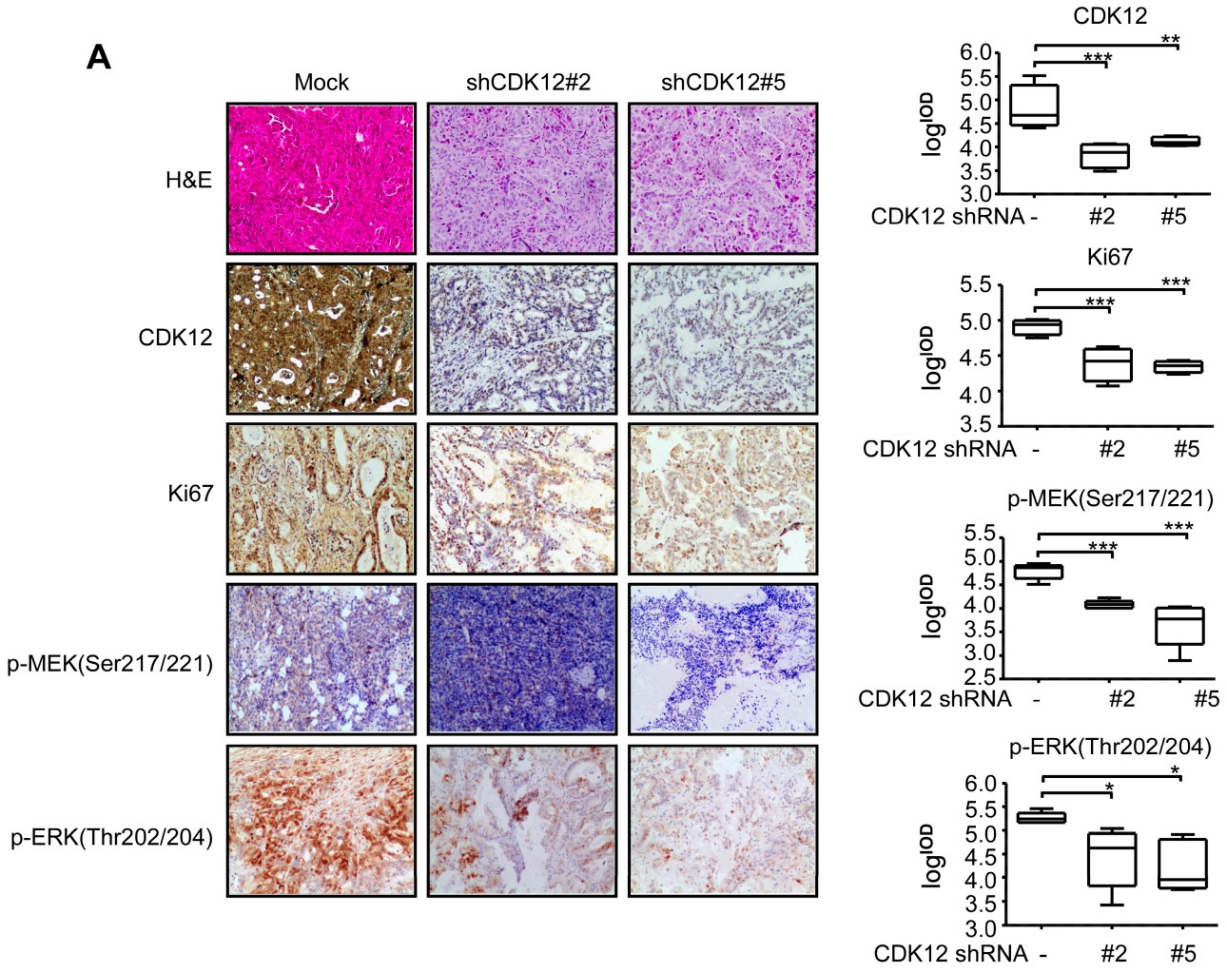
** CDK12 knocking down inhibits tumor growth by blocking MAPK signaling pathway.** A. Representative images and histogram of H&E and CDK12, Ki67, phospho-MEK, phospho-ERK IHC staining after formalin-fixed paraffin-embedded SNU-1 xenografts and PDXs tumors. Data represent means ±SD. P<0.05: *, p<0.01: **, p<0.001: ***. Significance determined by two-tailed Student's t test.

To elucidate the functional role of CDK12, cell cycle was analyzed after knock-down of the protein. Knock down of CDK12 induced cell cycle arrest at G2 phase (Figure 2A). To assess the effect of CDK12 in the growth of human gastric cancer, we utilized a cell xenograft animal model in NU/NU mice with CDK12 stable knock-down SNU-1 cells (2 × 10^6^) with shMock, shCDK12#2 and shCDK12#5. While shMock tumors became visible at Week 2, shCDK12 tumors started to show up at Week 4. Tumor size and weight were significantly lower in shCDK12 tumors compared with shMock group, and none of the shCDK12 tumors grew more than 1000 mm^3^ at the endpoint (Figure 2B). This result was recapitulated in two different cases of patient-derived xenograft (PDX) mouse models using lentivirus-mediated CDK12 knock-down (Figure 2C). Tumor tissues were also subjected to H&E staining and IHC staining with CDK12, Ki67, phospho-MEK and phospho-ERK. H&E staining images illustrated that knock-down of CDK12 suppressed the vitality of cancer cells, and IHC analyses showed that CDK12, Ki67, phospho-MEK and phospho-ERK levels were significantly decreased (Figure 3A) in CDK12 knock-down tumors. These data suggest that CDK12 drives human gastric cancer cell proliferation and tumor growth via MAPK signaling pathway, and that CDK12 may act as an effective therapeutic target in human gastric cancer.

### CDK12 interacts and co-localizes with PAK2 in human gastric cancer

**Figure 4:**
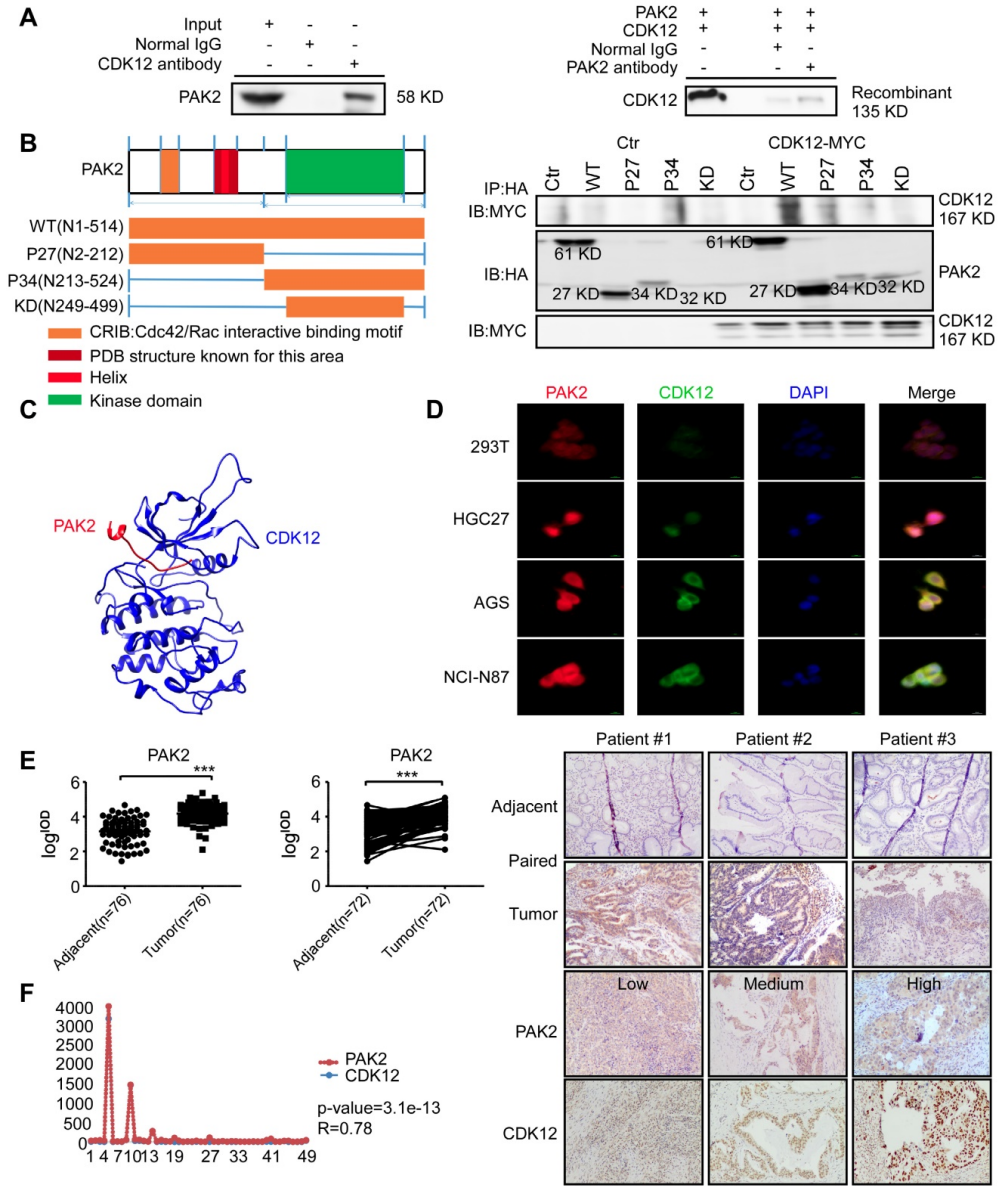
** CDK12 interacts with PAK2 and positively correlate with each other.** A. SNU-1 cell lysates and recombinant CDK12 and PAK2 proteins were immunoprecipitated with IgG or anti-CDK12/anti-PAK2 antibodies. Immunoprecipitated samples were subjected to western blotting with the indicated antibodies to detect the interaction of CDK12 and PAK2 *ex vivo* and *in vitro*. B. The domain architecture of human PAK2 (right) contains an N-terminal Cdc42/Rac interactive binding motif (CRIB) and kinase domain (aa249 to aa499. PAK2 can be cleaved into PAK2-p27 (aa2 to aa212) and PAK2-p34 (aa213 to aa524). (Left) HEK293T cells were transfected with MYC-tagged CDK12 and HA-tagged WT or N-terminally truncated PAK2 including p27, p34, and kinase domain. Immunoprecipitation was performed using anti-HA affinity gel followed by western blots using anti-MYC antibody. The PAK2-p27 binding to CDK12 is essential for this protein-protein interaction. C. The interaction between CDK12 (blue) and PAK2 (red) was predicted using a computational docking model. D. Representative images show that CDK12 (green) and PAK2 (red) are positively co-localized in nucleus (DAPI: blue) and cytoplasm shown by laser scanning confocal microscope through an immunofluorescence assay. E. The expression of PAK2 was examined by IHC staining with a gastric tumor array. The left panels show quantification of all samples and paired samples (n=72). The right panels show representative photographs of each group of paired samples. 100 × magnification. F. The IOD value statistics of CDK12 and PAK2 in human gastric tumor/adjacent tissues with IHC is shown in scatter diagram. CDK12 is positively correlated with PAK2 in human gastric tumors. The representative images are shown to the right from the same patient tissue. The R value calculated by Pearson formula. Data represent means ±SD. P<0.05: *, p<0.01: **, p<0.001: ***. Significance determined by two-tailed Student's t test.

We next investigated the underlying mechanism of CDK12-induced tumor progression. Pull down assay followed by mass spectrometry (MS) was performed to identify the potential CDK12 binding partners. We obtained 289 proteins in CDK12 pull down samples (Figure S1A). The analysis of protein-protein interaction network by Meta scape (Figure S1B) revealed promising target proteins including EGFR, MAP2K2, HK1, PAK2, RAD50 and SLK. By analyzing the correlation of these proteins with CDK12 on GEPIA data set, we found that PAK2 has a positive correlation with CDK12 (R=0.82, Figure S1C). Then, we examined the co-localization of CDK12 and PAK2 by laser scanning confocal microscope which revealed that they co-localized in the nuclear and cytoplasm in gastric cancer cells (Figure 4D). Next, we validated the interaction between CDK12 and PAK2 by co-immunoprecipitation experiments with both SNU-1 cell lysates and recombinant proteins (Figure 4A). We designed truncated PAK2 to identify the structural basis for CDK12-PAK2 interaction. PAK2 contains an N-terminal Cdc42/Rac interactive binding motif (CRIB) and a kinase domain, and the protein can be cleaved into two forms: PAK2-p27, PAK2-p34 34. A small portion of residues (N121-136) were annotated on PDB bank (Figure 4B, left panel). To determine the key region critical for CDK12-PAK2 interaction, we performed co-immunoprecipitation of MYC-tagged CDK12 with truncated HA-tagged PAK2 proteins over-expressed in HEK-293T cells.

We found that the PAK2-p27 fragment (N2-212) is essential for CDK12 binding to PAK2 (Figure 4B, right panel). Based on this result, we confirmed the interaction with a computational docking model of CDK12 and PAK2 (Figure 4C). These results together suggest that CDK12 and PAK2 are co-localized and bind with each other. Then, we used IHC staining with a PAK2 antibody on a patient tumor array that consisted of 76 adjacent tissues and 76 tumor tissues (72 paired tissues). PAK2 expression was significantly higher in tumor tissues compared with adjacent tissues (Figure 4E). Correlation of CDK12 and PAK2 in same patient tissue was analyzed by Pearson analysis, and the scatter diagram showed good positively correlation of CDK12 and PAK2 in patient tissues (Figure 4F). Thus, CDK12 interacts with, and is positively co-related to, PAK2.

### PAK2 is required for human gastric cancer cell proliferation and tumor growth

**Figure 5:**
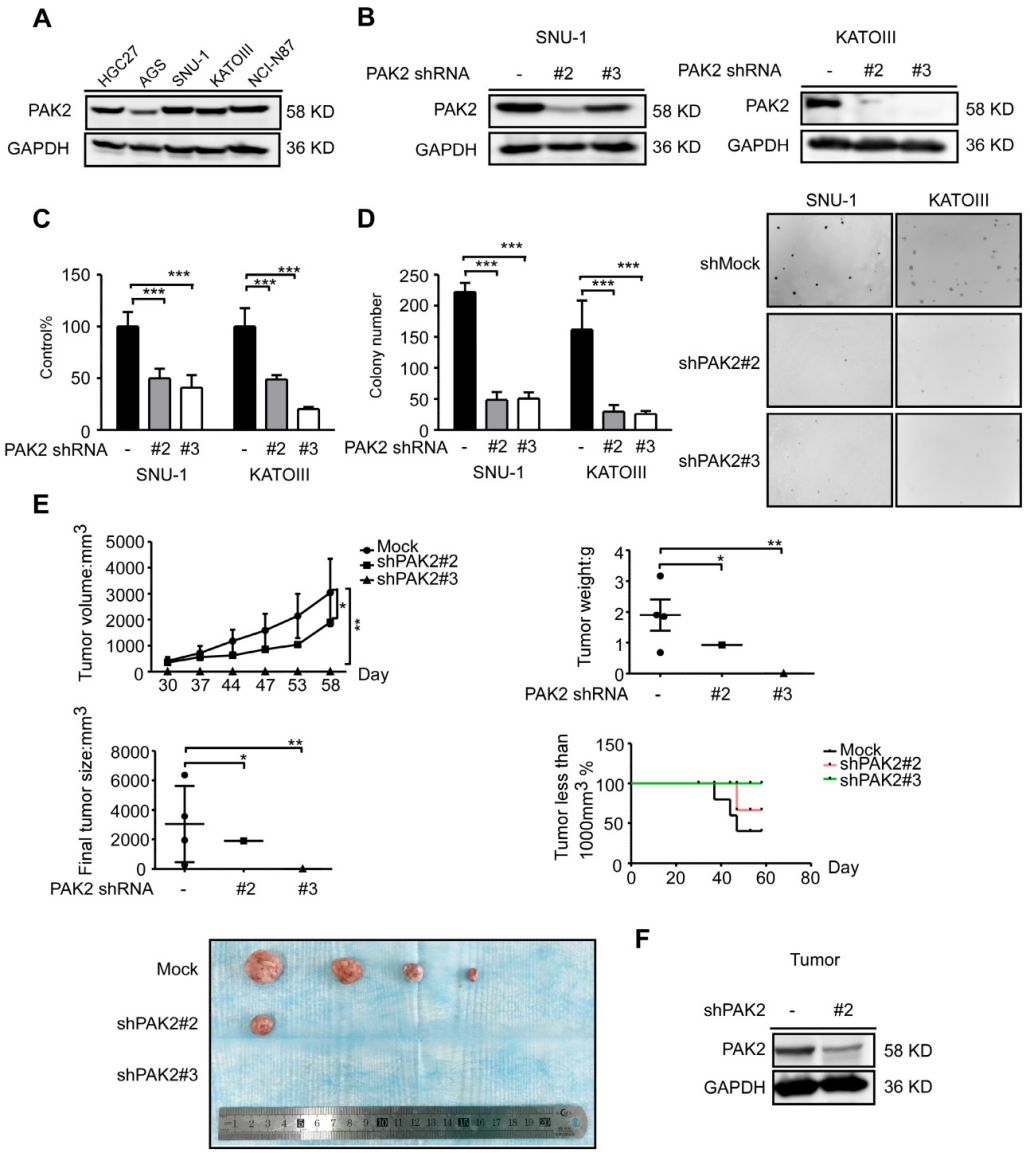
** PAK2 act as an oncogene in human gastric cancer.** A. The expression level of PAK2 in human gastric cancer cell lines was evaluated by western blotting. B. The level of PAK2 after knock-down by lentiviral transduction (shMock, shPAK2#2 and shPAK2#3) was measured by western blotting in SNU-1 and KATOIII cells. C. Cell viability after PAK2 knock-down was assessed by MTT (72 h after seeding cells) assay. D. Colony number of PAK2 knocking down cells was measured by anchorage-independent colony formation assay. The representative images are shown. E. Longitudinal tumor volume, tumor weight, final tumor size and percentage of mice with tumors less than 1000 mm^3^ at the endpoint were measured from shMock, shPAK2#2 and shPAK2#3 treated tumors of SNU-1. Tumor size was measured every 5-7 days, and the volume was calculated by V= (length) × (width) × (height). F. The effect of PAK2 knock-down in tumors was examined by WB. The representative images are shown. Data represent means ±SD. P<0.05: *, p<0.01: **, p<0.001: ***. Significance determined by two-tailed Student's t test.

To validate the function of PAK2 in gastric cancer, PAK2 expression level in human gastric cancer cell lines was verified by western blots analysis, and it was found to have a similar expression pattern as CDK12 (Figure 5A and Figure 1B). We constructed lentivirus targeting PAK2 in SNU-1 and KATOIII cells (Figure 5B). Cell viability and anchorage-independent colony growth were significantly inhibited by PAK2 knock-down (Figure 5C and 5D). This phenotype was also verified in SNU-1 xenograft NU/NU mice *in vivo*. Tumor size and tumor weight were significantly lower in PAK2 knock-down tumors. The number of mice with tumors less than 1000 mm^3^ was significantly higher in shPAK2 knock-down groups (Figure 5E-F). Therefore, these data suggest that a high level of PAK2 is associated with gastric cancer progression. The PAK2-CDK12 interaction may regulate important biological features in gastric cancer.

### CDK12 modulates tumor growth by phosphorylating PAK2 and activating MAPK signaling pathway

**Figure 6:**
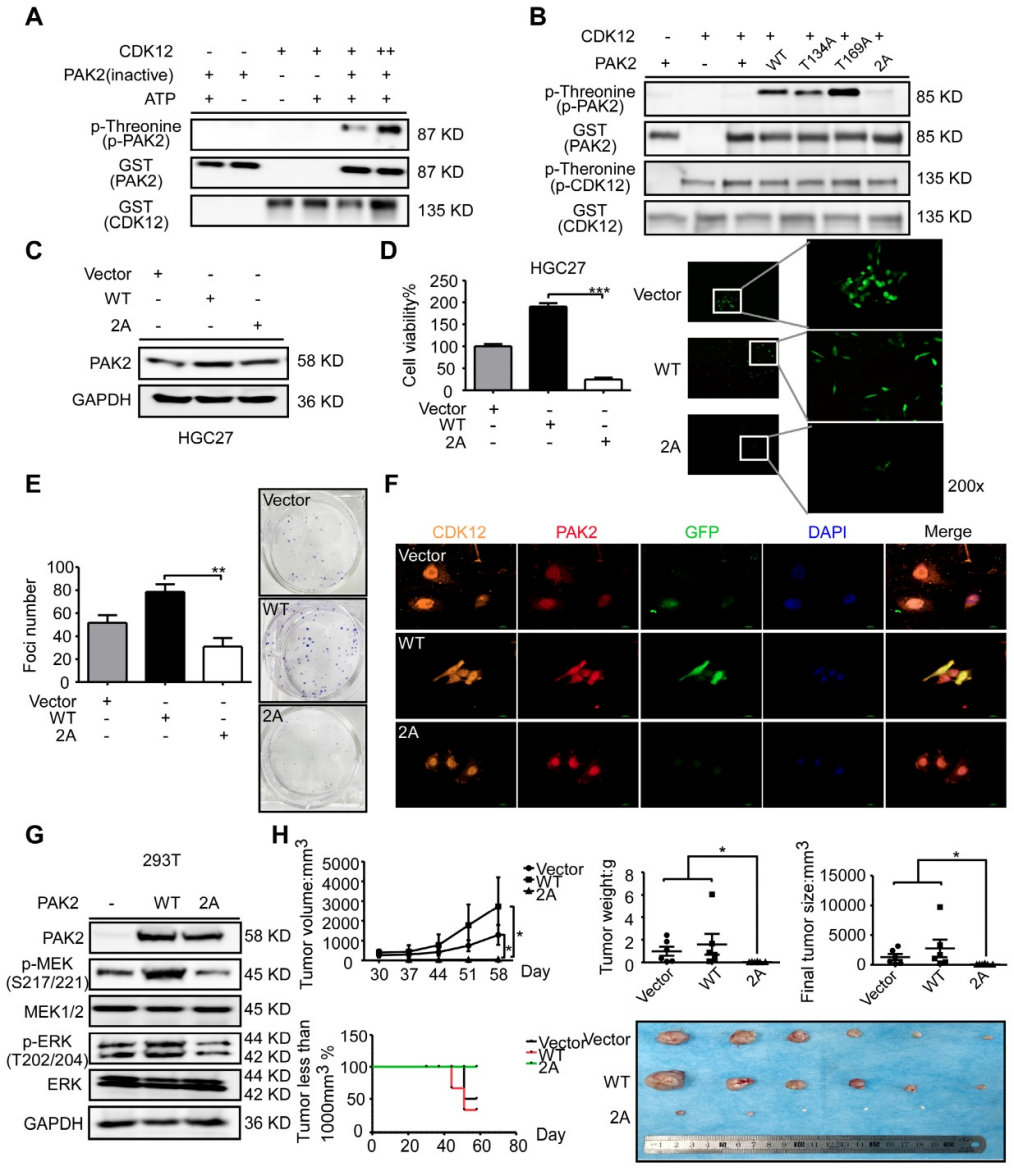
** CDK12 phosphorylates PAK2 at threonine 134/169 and activates MAPK signaling pathway.** A. Kinase assay of recombinant GST-tagged CDK12 with PAK2 *in vitro*. CDK12 (200 ng) and inactive PAK2 (400 ng) were mixed with ATP in kinase buffer at 30 ℃ for 30 min. Phospho-Threonine and GST were detected by western blots. B. Kinase assay of mutated PAK2 proteins (WT, T134A, T169A, 2A-T134A/T169A) with CDK12. Phospho-Threonine and GST were detected by western blots. C. Protein level of PAK2 was measured by western blotting after GFP-tagged lentiviral transfection of different mutation type (Vector, WT, 2A) of PAK2 in HGC27 cells. D. Cell viability of different mutation type (Vector, WT, 2A) PAK2 transfected cells was measured by MTT assay (72 h) and representative fluorescence of GFP are shown. E. Cell proliferation of different mutation type (Vector, WT, 2A) PAK2 transfected cells was determined by crystal violet foci formation assay and representative images are shown. F. Co-localization of CDK12 (orange), PAK2 (Vector, WT, 2A, red) and GFP (green) were measured by immunofluorescence after stable overexpression of PAK2 in HGC27. CDK12 and PAK2-WT are evenly distributed throughout the nucleus (DAPI: blue) and cytoplasm, while PAK2-V and PAK2-2A with CDK12 were mainly in nucleus. Representative images are shown. G. MAPK signaling pathway analysis by western blotting. Phospho-MEK and Phospho-ERK was measured by western blots in HEK293T cells with empty vector, PAK2-WT, or PAK2-2A transfected. Representative results are shown. H. Longitudinal tumor volume, tumor weight, final tumor size and percentage of mice with tumors less than 1000 mm^3^ at the endpoint were measured from empty vector, CDK12-WT, and CDK12-2A-overexpressed tumors of HGC27 in a xenograft model. Tumor size was measured every 5-7 days, the volume was calculated by V = (length) × (width) × (height). Tumors in each group are shown. Data represent means ±SD. P<0.05: *, p<0.01: **, p<0.001: ***. Significance determined by two-tailed Student's t test.

To further investigate the relationship between CDK12 and PAK2 in tumor growth, we utilized GPS, a kinase-specific phosphorylation prediction tool, to find that CDK12 may phosphorylate PAK2. After conducting mass spectrometry with CDK12-PAK2 kinase assay samples, we found two phosphorylation sites of PAK2: threonine 134 (T134) and threonine 169 (T169) (Figure S1D). These two sites are found only in PAK2 among the PAK family members, and the phosphorylation sites share a conserved motif between different species (Figure S1E). We verified the phosphorylation status by kinase assay with CDK12 and inactive PAK2 (Figure 6A).

To confirm these two sites, we constructed and purified the recombinant site mutation proteins (Figure S1F). The *in vitro* kinase assay with these purified proteins revealed that CDK12 cannot phosphorylate PAK2 T134A/T169A mutant type (PAK2-2A; Figure 6B). Then, we sought to validate the importance of these two phosphorylation sites via MTT assay and crystal violet foci assay after getting stable GFP-tagged PAK2 overexpression cells (Figure 6C). The result showed an inhibition of proliferation and colony formation by PAK2-2A in HGC27 cells (Figure 6D-E). Immunofluorescence assay by laser scanning confocal microscope showed that CDK12 and PAK2 are co-localized in nuclear and cytoplasm in PAK2 overexpression group, but the proteins are mainly in nuclear in vehicle and double sites mutation group (Figure 6F). Next, we tested if CDK12 induces tumor growth by activating PAK2-induced MAPK signaling pathway. MAPK signaling pathway key proteins, including phospho-MEK and phospho-ERK, were detected in different types of PAK2 cells (Vector, WT, 2A) by western blot analysis (Figure 6G). We found that the phosphorylation levels of MEK and ERK were significantly inhibited in PAK2 double mutant cells. The result was consistent with our hypothesis that the double mutation blocked the MAPK signaling pathway (Figure 3A). This phenomena was validated in HGC27 xenograft NU/NU mice model, showing that the tumors in double sites mutation group (2A) became visible later and grew more slowly than that of the wildtype group (Figure 6H). Taken together, CDK12 phosphorylates PAK2 at T134/T169 and activates MAPK signaling pathway speeding up cancer cell proliferation and tumor growth.

### Procaterol is a CDK12 inhibitor

**Figure 7:**
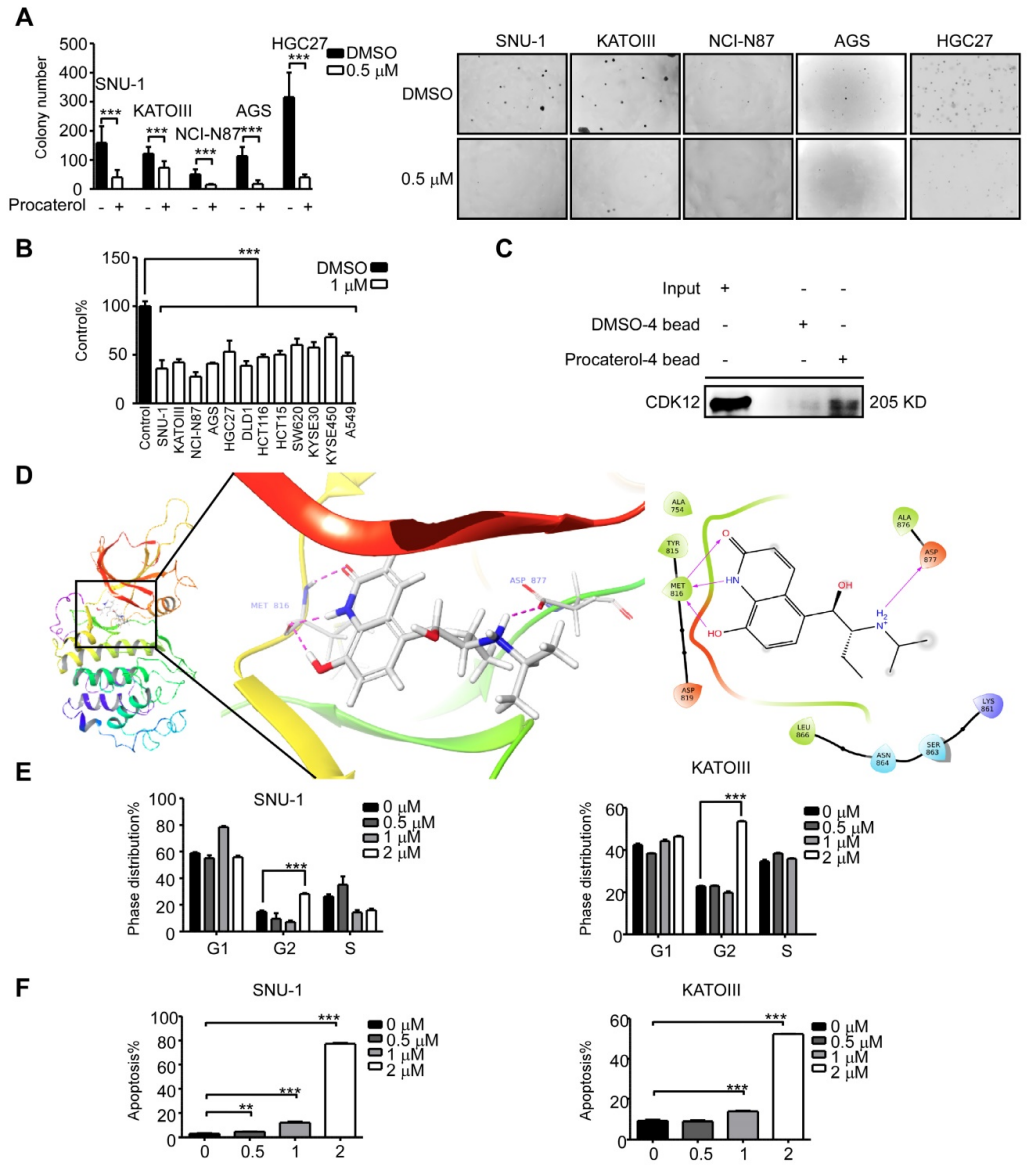
** Procaterol is a potent CDK12 inhibitor.** A. Colony formation of gastric cancer cells with vehicle control and procaterol (0.5 μM) treatment. Representative images are shown. B. Cell viability in different types of human cancer (gastric cancer, colon cancer, esophageal cancer, and lung cancer) cell lines with vehicle control and procaterol (1 μM) treatment, the representative result of 72 h are shown. C. The binding of procaterol to CDK12 in SNU-1 cell lysates was determined using sepharose 4B and procaterol-conjugated sepharose 4B beads. D. The interaction between procaterol and CDK12 was predicted by a computational docking model. (Left) The representative images show that procaterol binds with CDK12 at kinase responsible site (ASP877) and nucleotide binding site (MET816). (Right) Ligand interaction diagram of procaterol bind with CDK12. E. The effect of procaterol on cell cycle progression of gastric cancer cells. Cells were treated with 0.5, 1 or 2 μM of procaterol and then incubated for 48 h for cell cycle analysis. F. The effect of procaterol on apoptosis in gastric cancer cells. Cells were treated with 0.5, 1 or 2 μM of procaterol and then incubated for 72 h for the Annexin-V staining assay. Representative images are shown. Data represent means ±SD. P<0.05: *, p<0.01: **, p<0.001: ***. Significance determined by two-tailed Student's t test.

Until now, there are no Food and Drug Administration (FDA)-approved clinical CDK12 inhibitors as therapeutic drugs against diseases. We thus sought to find a CDK12 inhibitor by a computational docking model using the FDA-approved drug database. We chose 20 compounds with the highest docking score and tested their effects on human gastric cancer cells. We discovered that procaterol, a clinically used drug as β_2_-receptor agonist against bronchitis, has a dramatic effect on inhibiting cell viability and colony formation of gastric cancer cell lines, as well as colon cancer cells, lung cancer cells and esophageal squamous cell carcinoma cells (Figure 7A-B); in addition, we initially assessed the effects of valrubicin on gastric cancer cell viability (Figure S2A). Further, we showed procaterol could bind to CDK12 in SNU-1 cell lysates (Figure 7C). A computational docking model showed that procaterol directly binds to the CDK12 kinase activity responsible site ASP877 and nucleotide binding site MET816 residues (Figure 7D). *In vitro,* the CDK12 kinase assay using MBP or PAK2 as substrates verified that procaterol can directly inhibit the kinase activity of CDK12 (Figure S2B). Overall, we found that procaterol can serve as a CDK12 inhibitor, and the drug could induce cell cycle arrest and apoptosis (Figure 7E-F).

### *In vivo* efficacy of procaterol in patient-derived gastric cancer xenografts

**Figure 8:**
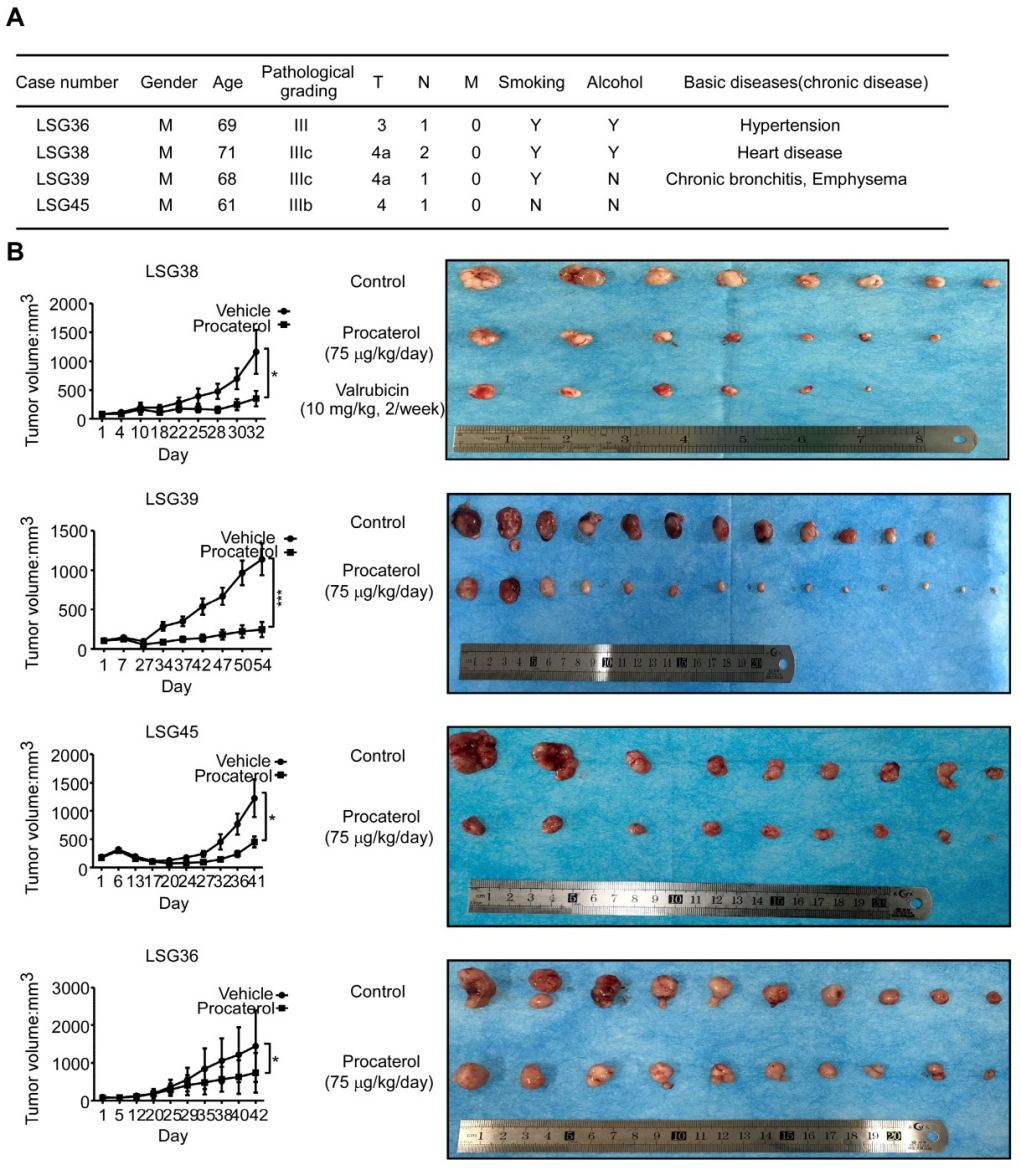
** Procaterol inhibits gastric tumor growth in PDXs.** A. Patient information of different PDX cases. Gender, age, pathological grade, clinical phases, smoking history, alcohol history and basic diseases are shown. B. Effects procaterol (75 μg/kg/day) administered by gavage. Tumor volumes of four different cases of PDX models were recorded. Tumors in each case are shown. Data represent means ±SD. P<0.05: *, p<0.01: **, p<0.001: ***. Significance determined by two-tailed Student's t test.

**Figure 9:**
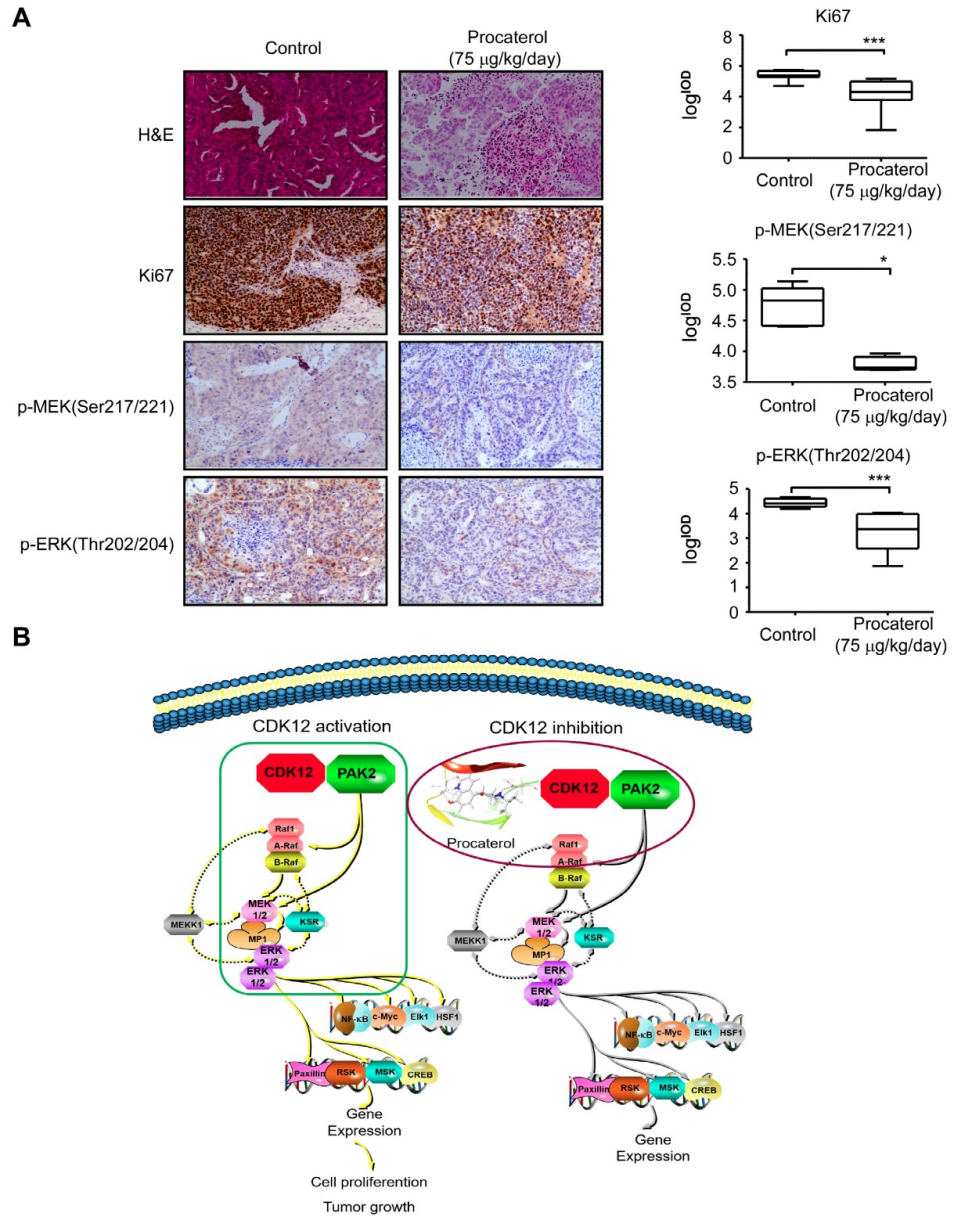
** Procaterol inhibits gastric tumor growth via MAPK signaling pathway verified in PDXs tumors.** A. H&E staining and IHC staining of Ki67, p-MEK, p-ERK in PDX tumor tissues. Representative images are shown. 100 × magnification. B. Proposed schematic diagram of CDK12 in whole study. CDK12 binds with and phosphorylates PAK2 at Threonine134/169 to activate MAPK signaling pathway such as p-MEK and p-ERK. This leads to the acceleration of cell proliferation and tumor growth in gastric tumor with high level of CDK12 (left). CDK12 inhibition by procaterol suppresses PAK2 phosphorylation and MAPK signaling (right). Data represent means ±SD. P<0.05: *, p<0.01: **, p<0.001: ***. Significance determined by two-tailed Student's t test.

Finally, we examined the effects of procaterol on four different cases of patient-derived gastric tumor xenografts (Figure 8A). All the cases showed a significant suppression of tumor growth by procaterol, especially in the CDK12 high expression level case LSG39, LSG38, LSG45 compared to the relatively low level of CDK12 LSG36, without demonstrating significant toxicity (Figure 8B, S3A). H&E staining revealed procaterol-induced tumor necrosis, and IHC staining of Ki67 and phospho-MEK, phospho-ERK indicated significant decrease of the biomarkers in procaterol-treated tumors compared to the vehicle group (Figure 9A).

## Discussion

Gastric cancer is a malignant cancer type that lacks valid therapeutics for late stage treatment. The cell cycle is a highly conserved and tightly adjusted biological process that regulates the proliferation and differentiation of different cells. Cell cycle related proteins, CDKs and cyclins, are important for the regulation of cellular proliferation and division. Genetic alterations, such as the overexpression of CDKs or cyclins, lead to a disordered cell-cycle progression in many cancer types including gastric cancer 35-37. Accordingly, high level of CDKs and cyclins are often correlated to a poor prognosis and undesirable outcome 38. Based on the importance of CDKs in tumorigenesis, there is an urgent need for the development of CDKs inhibitors for use as an effective cancer therapy. In this study, we identified CDK12 as an important therapeutic target for human gastric cancer. Our results elucidated a mechanistic basis by which CDK12 drives tumor progression in gastric cancer. We found that CDK12 is upregulated in human gastric cancer, and that high expression level of CDK12 is related with a poor overall survival 39. Down-regulation of CDK12 dramatically decrease gastric cancer cell proliferation and colony formation. Further, we validated this conclusion in cell line xenograft mouse models and patient-derived xenograft mouse models. To explore the molecular mechanisms, we conducted MS/MS with *in vivo* CDK12 immunoprecipitation assay and retrieved a list of candidate proteins. We found PAK2 is a downstream substrate of CDK12. CDK12 directly binds with and phosphorylates PAK2 at T134/T169, and thus activates MAPK signaling pathway that facilitates cell proliferation and tumor growth. Moreover, we found that procaterol directly binds and inhibits kinase activity of CDK12, and suppresses cancer cell proliferation and tumor growth *in vivo* (Figure 9B).

In contrast to other CDKs that plays a role in transcription, DNA damage/repair, or genomic stability by phosphorylating a series of CTD substrates 40, 41, we provided strong evidences that CDK12 phosphorylates PAK2, a non-CTD protein, and activates MAPK signaling pathway to promote cancer progression. Our results showed that the T134/T169 phosphorylated by CDK12 is important for the function of PAK2 in human gastric cancer. These phosphorylation sites of PAK2 may act as prognostic markers for gastric cancer cell proliferation and tumor growth. With this, we filled in the blanks that CDK12 phosphorylates a non-CTD protein with a detailed molecular mechanism.

From a clinical perspective, our data supports that CDK12 could serve as a potential biomarker and infusive therapeutic target for patients with high CDK12 expression levels. More importantly, we found that procaterol is an effective CDK12 inhibitor *in vitro* and *in vivo*. We used procaterol hydrochloride oral solution in the PDX experiments and obtained the compelling effects. This drug could be exploited in future clinical trials to confirm the dosage, safety, side effects and treatment conditions for anti-CDK12 therapy in clinic 42.

## Materials and methods

### Immunohistochemistry staining (IHC) and hematoxylin - eosin (H&E) staining

Tumor tissues were prepared for IHC and H&E analysis. The slides were baked 2 h and 40 min at 65 °C IHC and H&E, respectively. After de-paraffinization and hydration, the slides were boiled in citrate buffer for 90 s at a high temperature and pressure. Slides were then treated with H_2_O_2_ for 5 min, and incubated with primary antibody at 4 °C overnight. After incubation with a secondary antibody, slides were stained with DAB (3, 3'-diaminobenzidine). The immunohistochemistry staining was quantitated by calculating the integrated optical density (IOD) value measured by Image-Pro Plus analysis, and shown by log^IOD^ in figures.

### Cell lines

Human gastric cancer cell lines :AGS, SNU-1 and KATOIII were purchased from the Type Culture Collection of the Chinese Academy of Sciences (CAS, Shanghai, China), NCI-N87 and HGC27 were purchased from ATCC. All cell lines were cytogenetically tested and authenticated before use. The medium for AGS was F12K and the rest were cultured in RPMI-1640.

### Cell assays: cell viability assay, colony formation assay, foci assay, cell cycle and apoptosis analysis

For cell assays, cells were seeded as 5 × 10^3^ cells/well in 96-well plates and incubated for different time (0 h, 24 h, 48 h, 72 h) to measure cell proliferation by MTT assay. For checking the drug effect, after seeding cells overnight, cells were treated with various concentrations of compound or vehicle. For anchorage-independent colony formation assay, cells (8 × 10^3^ cells/well) were suspended in complete medium with 0.3% agar and different concentrations of compound with vehicle in a top layer over a bottom layer of 0.5% agar with vehicle and different concentrations of drugs in 6-well plates. The plates were maintained in CO_2_ incubator for 1 to 2 weeks. For foci forming assay, 300-900 cells were seeded in 6-well plates and treated with vehicle or compound for 10 to 14 days, then the foci was stained with 0.5% crystal violet. For cell cycle analysis, vehicle cells or stable knock down cells (4 × 10^5^) were seeded in 6 cm dishes, no treatment or treated with different concentrations of procaterol for 48 h or 72 h. Cells were fixed in 70% ethanol at -20 °C for at least 24 h, staining with propidium iodide then detected the cell cycle by flow cytometry. For the apoptosis assay, cells were stained with Annexin-V, and then cells were analyzed by flow cytometer.

### RNAi

For protein knock down and PAK2 site mutation overexpression, different lentivirus carrying shRNAs were produced by transfecting lenti-293T with pLKO.1 encoding shRNA, pMD2.G and psPAX2. Cells were selected by puromycin (2 μg/mL) after infection with lentivirus for 1 to 2 days. For transient transfection overexpression, plasmids were transfected into 293T or HGC27 and AGS by using Simple-Fect reagent as the ratio 1/2. Cells were harvested 24-48 h later.

### Immunoprecipitation

For immunoprecipitation assay, the same amount of human gastric cancer cell lysates were incubated with normal IgG or anti-CDK12 antibody or PAK2 antibody, rotating at 4 °C overnight. Then 50 μL of protein A/G agarose beads (Santa Cruz) was added in each samples and rotated at 4 °C for 2 h. After washing out of beads, PAK2 or CDK12 band was detected by western blot. For immunoprecipitation assay with truncated PAK2 and CDK12, truncated HA-PAK2 was transfected into HEK-293T separately or co-transfecting with MYC-CDK12, and cell lysates were incubated with normal IgG or anti-HA antibody, then detected the band of MYC tag by western blot.

### Computer docking model

The computer modeling of CDK12 with PAK2 and procaterol with CDK12 was performed by the Schrödinger Suite 2015 software programs. The crystal structures of CDK12 and PAK2 were downloaded from the Protein Data Bank (PDB), the PDB number of CDK12 is 5ACB, 3PCS for PAK2, and the drug bank number of procaterol is DB01366. All the modeling was prepared under the standard procedures of the Protein Preparation Wizard in Schrödinger 43.

### Pull down assay and mass-spectrometry

For pull down assay with SNU-1 cell lysates, after incubation with normal IgG or anti-CDK12 antibody, CDK12 complex was pulled down by Protein A/G beads. The protein complex was subjected to SDS/PAGE and each gel lane was digested with trypsin at 37 °C overnight in a shaker. Then the digested peptides were analyzed with a mass spectrometer 44-46. For pull down assay of procaterol with CDK12, DMSO or procaterol binds to 4B beads, and incubate with same amount of cancer cell lysates at 4 °C overnight with rotating, then detected the band of CDK12 by western blot.

### Kinase assay

For CDK12 and PAK2 kinase assay, proportional CDK12 and PAK2 was added with ATP in kinase buffer, the mixture was incubated for 30 min at 30 °C and the band was assessed by Western blot analysis.

### Immunofluorescence

For immunofluorescence, cells were seeded in 24 wells plates over glass cover slips, incubated cells with primary antibody of CDK12, PAK2 after fixed with 4% Paraformaldehyde (PFA) overnight. The next day add secondary antibody of different wave length with DAPI after washed 3 times. Then, fluorescence was observed by laser scanning confocal microscope (LSCM) and take photos.

### Vector construction, protein expression and purification

For protein purification, the pECE-M2-PAK2 plasmid was changed to the pGEX-6P-1 N-GST tagged vector and site mutation were cloned using Q5 enzyme by PCR. The plasmid was transfected into BL-21 for protein expression induced with IPTG at 37 °C for 4 h. Then, the protein was subjected to a pull down assay and purified by GST beads. The purified protein was stored at -80 °C for the kinase assay.

### Mouse xenografts

All institutional and national guidelines for the care and use of laboratory animals were followed. For cell xenografts NU/NU mice, 2 × 10^5^ - 2 × 10^6^ cells were injected subcutaneous, different groups in one mouse to remove individual differences (Mock: left foreleg, shRNA1: right foreleg, shRNA2: back). Tumor volumes were measured by Vernier caliper 3-7 d and calculated as V = (length) × (width) × (height). For patient-derived-xenografts (PDX), when tumors grew to an average volume approximately 200 mm^3^, mice were divided into different treatment groups according to tumor volume and body weight as follows: (1) mock group; (2) lentivirus group 1; (3) lentivirus group 2; (4) control group; (5) valrubicin treatment group; (6) procatrol treatment group. Lentivirus and valrubicin were injected into tumor directly, while procaterol was administered by gavage once a day. Mice were monitored until a tumor volume of 1000 mm^3^ was reached, mice were then sacrificed and tumors were extracted for further IHC and H&E analyses.

### Quantification and statistical analysis

Statistical analysis was performed by GraphPad Prism. Data shown is representative of multiple assays. Data with error bars represent mean ± standard deviation (SD). Statistical significance was based on the paired two-tailed Student's t test.

### Material resources

See in supplemental materials.

## Supplementary Material

Supplementary figures.

Key Resources Table.

## Figures and Tables

**Table 1:** Cohort characteristics of gastric cancer patient

Clinicopathological characteristics		Number (n=69)	Percent (%)	CDK12		p-value
				Low (n=34)	High (n=35)	
Age	Median (range)	64 (30-86)		60.5±10.72	69±10.73	0.0149(*)
Gender	Male	55	79.7	28 (82.4)	27 (77.1)	0.8535
	Female	14	20.3	6 (17.6)	8 (22.9)	
Pathology grade	Ⅱ	23	33.3	8 (23.5)	15 (42.9)	0.1622
	Ⅱ-Ⅲ	22	31.9	11 (32.4)	11 (31.4)	
	Ⅲ	24	34.8	15 (44.1)	9 (25.7)	
Clinical stage	1	4	5.9	1 (3.0)	3 (8.6)	0.7083
	2	17	24.6	8 (23.5)	9 (25.7)	
	3	41	59.4	20 (58.8)	21 (60.0)	
	4	7	10.1	5 (14.7)	2 (5.7)	
Pathomorphology	Ulcerative type	61	88.4	30 (88.2)	31 (88.6)	0.3345
	Protrude type	7	10.1	3 (8.8)	4 (11.4)	
	Infiltrating type	1	1.5	1 (3.0)	0 (0.0)	
Tumor size(mm^3^)	Median (range)	19.29 (3-234)		19.54±41.11	18±31.22	0.4460
Lymph node	Median (range)	25 (8-71)		24.5±11.27	27±7.24	0.2346
Positive lymph node	Median (range)	3 (0-42)		4±10.60	3±5.49	0.0869

## Abbreviations

CDK12: cyclin dependent kinase 12
PAK2: p21 activated kinase 2
CTD: C-terminal domain
MS: mass spectrometry
CDX: cell xenograft mouse model
PDX: patient-derived xenograft mouse model
