# Supplementary material for: CDK12 and PAK2 as novel therapeutic targets for human gastric cancer
Source: Theranostics. 2020 May 15;10(14):6201–15. doi: 10.7150/thno.46137 (PMC7255043; doi:10.7150/thno.46137)
Supplement: Supplementary file 1 — Supplementary figures. [file thnov10p6201s1.pdf]

Supplemental figures

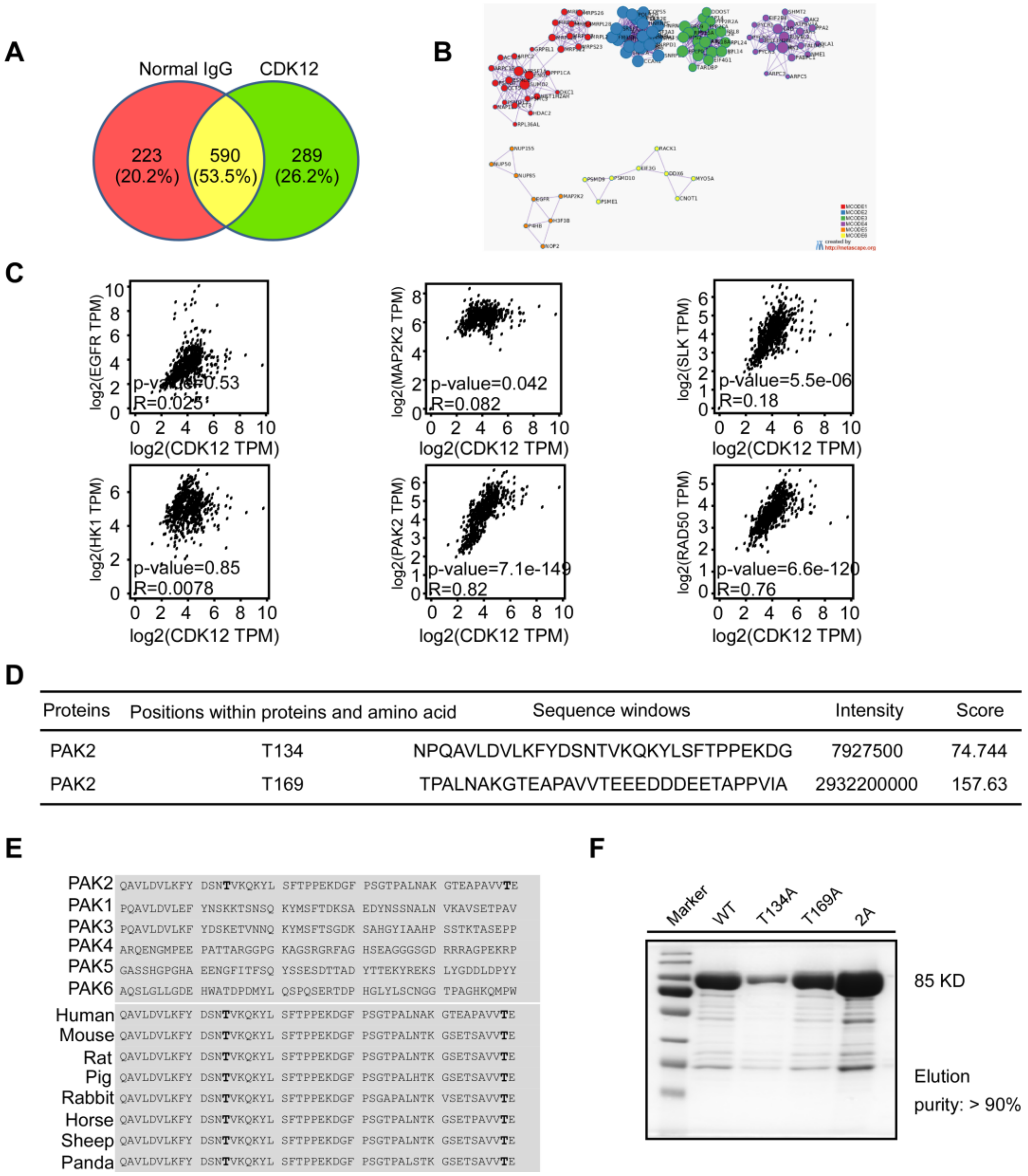

Figure S1. CDK12 interacts with and phosphorylates PAK2

A. Venn diagram (Venny 2.1) of potential CDK12 interact protein candidates (total 289). SNU-1 cell lysates were analyzed by mass spectrometry (MS/MS) after pull down assay incubated with normal rabbit IgG or anti-CDK12 antibody. The pulled-down samples were subjected to SDS-PAGE and cut into different pieces, digested with trypsin before MS/MS.

B. Protein-protein interaction network modes were clustered by Metascape software and the

representative modes are shown.

C. Potential CDK12 binding partners based on MS results analyzed on GEPIA data set.

D. MS/MS analysis revealed phosphorylation sites (T134/T169) of PAK2.

E. Sequence alignment of different PAK family members and protein sequence around the phosphorylation site motif across species.

F. Recombinant GST-tagged PAK2 (WT, T134A, T169A, 2A) proteins with different site mutation were purified after overexpression in *E.coli* using GST beads. The purified proteins washed in elution buffer and the purity was assessed through SDS-PAGE by Coomassie blue staining. The purity of both mutations more than 90%.

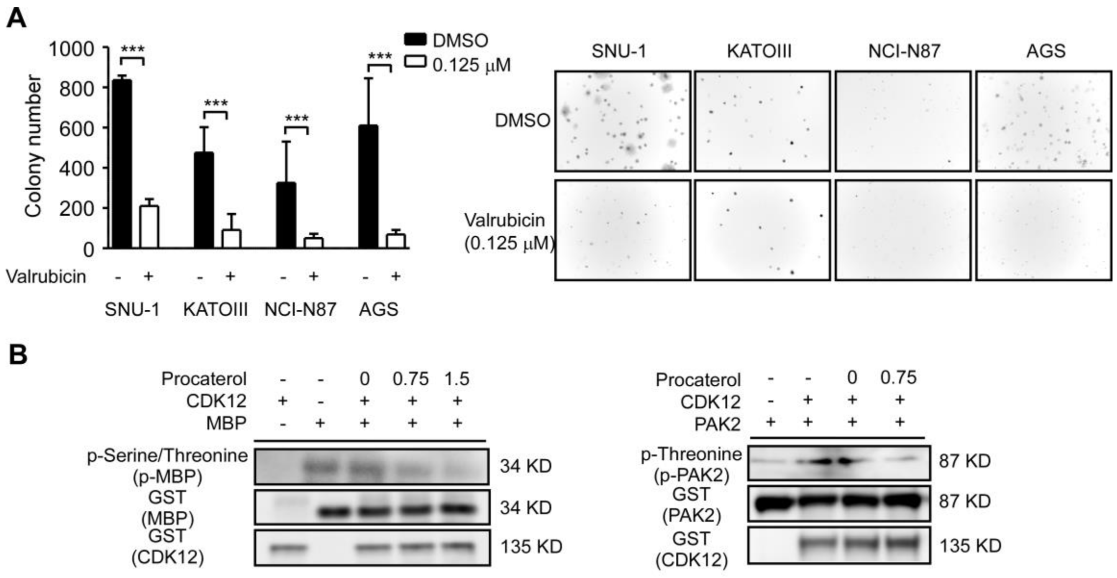

Figure S2. Procaterol inhibit CDK12 kinase activity

A. Cell viability of human gastric cancer cells treated with valrubicin (0.125 μM) was checked by anchorage-independent colony formation assay, representative images are shown.

B. The effect of procaterol on CDK12 activity was evaluated in an *in vitro* kinase assay using MBP and inactive PAK2 as substrates.

A

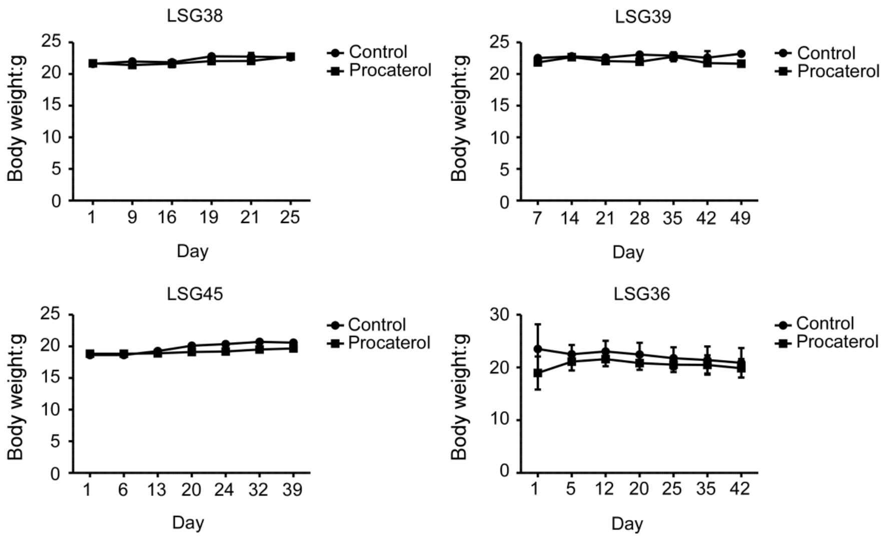

Figure S3. Procaterol shows no significant toxicity

A. The body weight of PDX mouse models.
